# The Fate of Tau Aggregates Between Clearance and Transmission

**DOI:** 10.3389/fnagi.2022.932541

**Published:** 2022-07-18

**Authors:** Assel Seitkazina, Kyu Hyeon Kim, Erin Fagan, Yoonsik Sung, Yun Kyung Kim, Sungsu Lim

**Affiliations:** ^1^Convergence Research Center for Brain Science, Brain Science Institute, Korea Institute of Science and Technology (KIST), Seoul, South Korea; ^2^Division of Bio-Medical Science and Technology, Korea Institute of Science and Technology (KIST) School, University of Science and Technology (UST), Seoul, South Korea; ^3^Department of Biological Engineering, Massachusetts Institute of Technology (MIT), Cambridge, MA, United States

**Keywords:** tau, tau clearance, tau transmission, tauopathy, pathological tau

## Abstract

Neuronal accumulation of mis-folded tau is the pathological hallmark of multiple neurodegenerative disorders, including Alzheimer’s disease. Distinct from amyloid plaques, which appear simultaneously throughout the brain, tau pathology develops first in a specific brain region and then propagates to neuroanatomically connected brain regions, exacerbating the disease. Due to the implication in disease progression, prevention of tau transmission is recognized as an important therapeutic strategy that can halt disease progression in the brain. Recently, accumulating studies have demonstrated diverse cellular mechanisms associated with cell-to-cell transmission of tau. Once transmitted, mis-folded tau species act as a prion-like seed for native tau aggregation in the recipient neuron. In this review, we summarize the diverse cellular mechanisms associated with the secretion and uptake of tau, and highlight tau-trafficking receptors, which mediate tau clearance or cell-to-cell tau transmission.

## Introduction

Tau is a microtubule-associated protein, which is extremely soluble in physiological condition (Barbier et al., 2019; Iwata et al., 2019). Full-length human tau contains a microtubule binding domain, which contain 18 lysine residues. The positive charges of lysine residues drive tau to bind a microtubule, a negatively charged polymer (Jho et al., 2010). Upon binding, tau stabilizes microtubules promoting microtubule assembly, which is critical for axonal outgrowth (Fischer et al., 2009; Rodríguez-Martín et al., 2013). Tau also plays a role in anchoring microtubules to other cytoskeletal filaments and cellular organelles for maintaining axonal structure and function (Miyata et al., 1986; Jung et al., 1993). More recent studies have elucidated new physiological roles of tau as a synaptic protein in accelerating spine formation, dendritic elongation, and synaptic plasticity (Chen et al., 2012; Kimura et al., 2014; Zempel et al., 2017; Velazquez et al., 2018). Despite the expected role of tau in neuronal structure and function, tau-deficient mice appeared to be normal presenting with no overt phenotype or malformations in the nervous system (Harada et al., 1994). Depletion of tau did not affect axonal elongation in cultured neurons (Tint et al., 1998). These observations suggest that other microtubule-associated proteins could play the role of tau in the absence of tau protein (Takei et al., 2000). While the reported tau knockout strains presented without obvious phenotype when young, one tau knockout strain showed muscle weakness and motor deficits when old, suggesting the pathological role of tau in age-related diseases (Ikegami et al., 2000; Lei et al., 2012).

**FIGURE 1:**
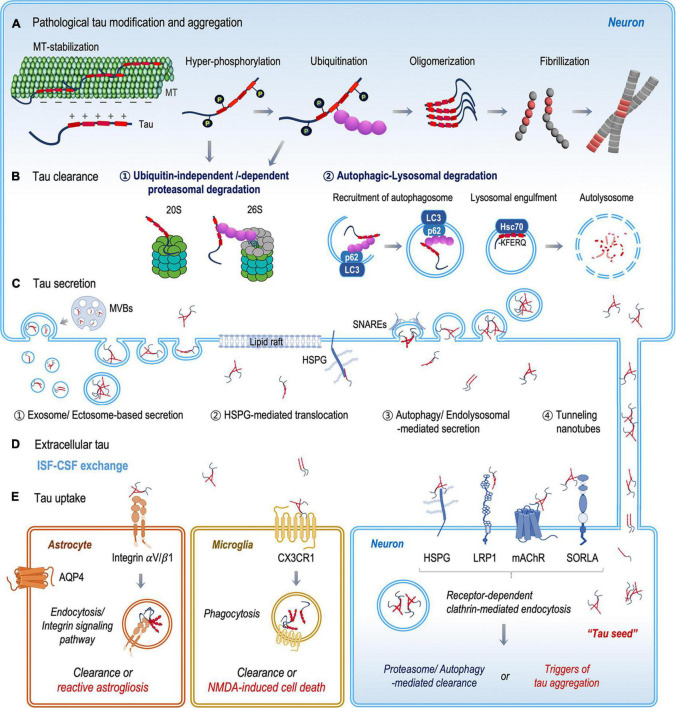
Schematic illustration of the fate of tau protein between clearance and transmission. **(A)** Mis-localized tau undergoes various post-translational modifications, such as phosphorylation and ubiquitination. Chemically and structurally modified tau becomes aggregated and accumulated in the cytoplasm. **(B)** Mis-folded and aggregated tau proteins are degraded by the ubiquitin–proteasome and autophagosome-lysosomal systems. **(C)** Tau proteins are released into the extracellular space both as a free protein and in vesicles, through (1) vesicular and (2–4) non-vesicular secretion pathways. **(D)** Extracellular tau proteins are eliminated through ISF–CSF exchange within the glymphatic system or are taken up by neurons, microglia, and astrocytes. **(E)** In recipient neurons, extracellular tau proteins bind specific tau receptors (HSPG, LRP1, mAChRs, and SORLA) and are internalized by receptor-dependent clathrin-mediated endocytosis. Extracellular tau clearance by microglia and astrocytes includes the binding and internalization of tau *via* the CX3CR1 receptor in microglia and the integrin αV/β1 receptor in astrocytes.

Under pathological conditions, tau hyperphosphorylation occurs, and the charge balance between tau and microtubules becomes disrupted (Gong et al., 2005; Alonso et al., 2018). Consequently, hyperphosphorylated tau dissociates from microtubules and accumulates in the cytosol (Figure 1A). In the cytoplasm, mis-localized tau undergoes various post-translational modifications, such as acetylation, sumoylation, ubiquitination, glycosylation, methylation, nitration, truncation, and inter-molecular bond formation (Martin et al., 2011; Haque et al., 2016). Tau acetylation prevents the degradation of phosphorylated tau (Min et al., 2010). Disulfide-bond formation between tau molecules generates structurally stable tau oligomers that play a critical role in tau aggregation and transmission (Kim et al., 2015; Prifti et al., 2021). Proteolytic cleavage of tau is also crucial in the generation of compact filaments by removing the N-and C-terminal flanking regions of tau (Friedhoff et al., 2000). The complex pattern of tau modifications would change the physical and chemical properties of tau as the infectious, aggregation-prone form. The accumulation of filamentous tau aggregates is a pathological hallmark of tauopathies, including Alzheimer’s disease (AD), frontotemporal dementia (FTDP-17), Pick’s disease, and progressive supranuclear palsy (PSP) (Iqbal et al., 2010; Orr et al., 2017). In tauopathy brains, tau pathology develops first in a specific brain region, and then propagates into anatomically connected brain regions. For example, in the brains of AD patients, tau pathology appears first in the trans-entorhinal cortex and propagates into the neuroanatomically connected hippocampus, causing cognitive impairments (Braak and Braak, 1991). In PSP brains, tau pathology develops in the basal ganglia at an early stage, causing motor symptoms, and later spreads to the cerebellum and brainstem, resulting in motor failure (Williams et al., 2007).

As a possible mechanism of tau propagation in the brain, a cell-to-cell transmission hypothesis has been raised. Tau transmission occurs through the trans-synaptic transfer of mis-folded tau aggregates from a donor cell to a recipient cell. The process can be divided into three basic steps: (1) secretion of pathological tau aggregates from donor cells, (2) uptake of the secreted tau species by recipient cells, and (3) initiation of tau pathology in the recipient cells (Gibbons et al., 2019; Brunello et al., 2020). Accumulating evidence has demonstrated that tau protein is actively released into the extracellular space in the brain. Higher levels of tau (453.6 ± 343.5 pg/mL) and tau phosphorylated at T181 residue (67.8 ± 18.0 pg/mL) were detected in cerebrospinal fluid (CSF) of AD patients (Haense et al., 2008; Kandimalla et al., 2013). Phosphorylated tau at T181, T217, and T205 residues were detected in the CSF of AD patients, and the levels of phosphorylated tau increased during the disease progression, positively correlating with neuronal dysfunction (Fagan et al., 2007; Barthélemy et al., 2020). Increased levels of N-224 tau fragments were also observed in the CSF of AD patients and in conditioned medium of neuron cell cultures (Ramcharitar et al., 2013; Zhang et al., 2014; Cicognola et al., 2019). Although transmittable tau species were not fully characterized, several recent studies have shown that phosphorylated tau can be translocated through the plasma membrane (Katsinelos et al., 2018; Merezhko et al., 2018). Also, various forms of tau aggregates were detected in the extracellular space (Lasagna-Reeves et al., 2011; Goedert, 2016). Significantly increased levels of tau oligomers and a mix of dimers, trimers, and tetramers were detected in the CSF of AD patients (Takeda et al., 2015; Sengupta et al., 2017). A line of evidence suggests that soluble tau oligomers, before forming fibrils, induce neuritic degeneration, perturb fast axonal transport of membranous organelles (Swanson et al., 2017), and increase reactive oxygen species leading to neuronal cell death (Lasagna-Reeves et al., 2012; Kim et al., 2015; Shafiei et al., 2017). In addition, intra-hippocampal injection of tau oligomers, rather than tau fibrils, were effective in inducing memory impairments in mice (Fá et al., 2016; Gerson et al., 2016a; Puzzo et al., 2017). Alongside neurotoxicity, tau oligomers were actively internalized into cells to a greater extent than tau monomers or insoluble aggregates, and the internalized tau oligomers could act as a structural seed to initiate native tau aggregation (Usenovic et al., 2015; Gerson et al., 2016b; Manassero et al., 2017; Swanson et al., 2017). The presence of extracellular tau has long been considered the result of neuronal cell death and the release of intracellular contents (Fricker et al., 2018). However, recent accumulating studies have reported extracellular tau also exists along with healthy and mature neurons (Pooler et al., 2013; Yamada et al., 2014; Wu et al., 2016). Upon stimulation of neuronal activity, tau binds to the cytosolic side of synaptic vesicles and is secreted with neurotransmitters (McInnes et al., 2018). An increased level of tau was detected in the interstitial fluid (ISF) of tau transgenic mice stimulated with optogenetic activation (Yamada et al., 2014b). Moreover, the treatment of extracellular tau induces neuronal hyperactivity leading to the secretion of endogenous tau suggesting the bidirectional relationship between neuronal activity and tau release (Bright et al., 2015).

While the amount and types of extracellular tau vary depending on the disease status, it is clear that tau is constantly secreted into the extracellular space (Wattmo et al., 2020). Then, adjacent neurons uptake extracellular tau species through diverse endocytosis mechanisms. In the recipient neurons, pathogenic tau aggregates can initiate tau pathology as a “structural seed” for native tau aggregation (Clavaguera et al., 2009, 2013; Lasagna-Reeves et al., 2012). In 2013, an *in vitro* model of tau propagation, in which somatodendritic and axonal compartments were separated in microfluidic chambers, showed that tau aggregates were internalized by both compartments and transported along the axon in either the anterograde or the retrograde direction (Wu et al., 2013). In 2014, an *in vivo* model of tau propagation demonstrated contralateral and anterior/posterior spread of tau pathology in the brain of tau-transgenic mice (Ahmed et al., 2014). In the brain, the spread of tau pathology was dependent on synaptic connectivity rather than spatial proximity. Accordingly, extracellular tau has emerged as an important target for tau-targeted immunotherapy. Toward this, efforts have been made to characterize prion-like tau species and tau trafficking-receptors, which are critical in tau transmission. A comprehensive understanding of tau transmission would provide new perspectives for the diagnosis and prevention of tau pathology. Here, we review the diverse cellular mechanisms associated with tau clearance and transmission.

## Molecular Mechanisms of Tau Secretion

In classical secretory pathways, secretory proteins contain signal peptides, which are well-known sequence motifs targeting proteins for translocation across the endoplasmic reticulum membrane (Cohen et al., 2020). Since tau protein does not contain an apparent signal peptide, tau was thought to be released from dying or dead neurons. However, proteins without a signal peptide can also be secreted into extracellular space *via* non-conventional pathways. Over the last decade, mounting evidence has demonstrated that tau secretion occurs through diverse non-conventional pathways. Due to the implication in tau pathology, extensive studies have investigated the pathological roles of the extracellular tau. In comparison, the physiological role of tau in the extracellular space remains largely unknown. A few preliminary studies suggested novel functions of tau in the extracellular space. For example, the treatment of extracellular tau increases the electrical activity of primary neuron culture (Bright et al., 2015) and the treatment of tau fibrils increased the formation of tunneling nanotubes (TNTs) between neurons (Tardivel et al., 2016). Although the role of tau is not clear, tau proteins are actively secreted into the extracellular space in both physiological and disease conditions. In this section, we will review the diverse non-conventional mechanisms associated with tau secretion (Figures 1B,C).

### Heparan Sulfate Proteoglycan-Mediated Translocation of Tau

Tau translocation across the plasma membrane is recently being highlighted as a key mechanism of non-conventional tau secretion. Tau translocation is a two-step process: (1) recruitment of tau to the plasma membrane and (2) translocation mediated by heparan sulfate proteoglycans (HSPGs) (Katsinelos et al., 2018; Merezhko et al., 2018). Lipid microdomains containing cholesterol, sphingomyelin, phosphatidyl serine, and PI(4,5)P2 are the major trans-elements responsible for recruiting tau to the plasma membrane. Katsinelos et al. (2018) showed that PI(4,5)P2 could initiate the translocation of tau. In their study, lysine residues in microtubule-binding domain of tau can form an interaction surface with the negatively charged PI(4,5)P2 on the plasma membrane. Lipid rafts containing cholesterol and sphingomyelin are necessary for the recruitment of pathological tau aggregates (Lasagna-Reeves et al., 2014; Patel et al., 2015). Merezhko et al. (2018) demonstrated that the disruption of lipid rafts in the plasma membrane decreased tau secretion by 90%. Once tau is recruited to plasma membrane, HSPG-mediated translocation occurs. HSPGs are complex carbohydrate-modified proteins carrying heparan sulfate (HS) and are ubiquitously expressed on the transmembrane. HSPGs can recruit and cluster molecules in transmembrane domains, mediating their translocation (Condomitti and de Wit, 2018). Accumulating evidence suggests that HSPGs anchor tau and mediate bi-directional translocation of tau through the plasma membrane. Xu and Esko (2014) showed that a decrease of HSPGs in the plasma membrane suppressed tau secretion significantly. Altogether, tau protein can be secreted into the extracellular space *via* direct translocation through the plasma membrane.

### Autophagy/Endolysosomal-Mediated Secretion

In cells, proteins that are no longer needed or are mis-folded/damaged are degraded by the ubiquitin–proteasome system (UPS) and the autophagy-lysosome system (Wong and Cuervo, 2010; Dikic, 2017). In the early stage of tau pathology, mis-localized and mis-folded tau proteins start to accumulate in the cytosol. The UPS mediates the clearance of soluble tau monomers and aggregates. In the UPS, tau proteins are tagged with ubiquitin and the ubiquitinylated tau proteins are digested by the 26S proteasome (Schmidt and Finley, 2014). As tau pathology progresses, tau proteins assemble into large, insoluble fibrils, which exceed the capacity of the UPS and lead to proteasome dysfunction (Morishima-Kawashima et al., 1993). As a result, polyubiquitinated tau proteins are accumulated in the cytosol and become a compartment of insoluble tau aggregates such as paired helical filaments (Morishima-Kawashima et al., 1993).

As an alternative to the UPS, autophagy plays a critical role in the clearance of pathological tau aggregates. Depending on the cargoes, autophagy is divided into three types: (1) chaperon-mediated autophagy (CMA), (2) endosomal-microautophagy (e-MI), and (3) macroautophagy. Soluble tau monomers and oligomers are cleared by CMA and e-MI, whereas large, insoluble tau aggregates are cleared by macroautophagy (Wang et al., 2009; Krüger et al., 2012; Caballero et al., 2018; Uytterhoeven et al., 2018). In macroautophagy, tau aggregates are first wrapped inside a specialized organelle, called an autophagosome, and the autophagosomes fuse with lysosomal vesicles to form autolysosomes, enabling the degradation of their cargoes (Dikic and Elazar, 2018). However, in a degenerating neuron, the axonal supply of lysosomes is restricted, resulting in the accumulation of autophagosomes (Farfel-Becker et al., 2019). Recent accumulating evidence has demonstrated that the deficiency of lysosomes drives the autophagosomes to fuse with the plasma membrane, leading to the secretion of their cargos into the extracellular milieu (Tang et al., 2015; Lonati et al., 2018; Kang et al., 2019). Tang et al. (2015) reported electron microscopic images of autophagic vacuoles containing tau approaching the plasma membrane. The fusion of autophagosomes with the plasma membrane is mediated by the assembly of soluble *N*-ethyl maleimide sensitive factor attachment protein receptors (SNAREs) (Wang et al., 2016). SNARE proteins (Sec22b) on autophagosomes interact with SNAREs (syntaxin 3/4 and SNAP-23/29) on the plasma membrane for membrane fusion and cargo secretion (Kimura et al., 2017a,b). CMA is also involved in the clearance and secretion of soluble tau monomers and oligomers. Tau protein contains KFERQ-like motifs, known as CMA-substrates, which are recognized by heat shock cognate 71 kDa protein (Hsc70) (Wang et al., 2009; Robert et al., 2019). Hsc70 mediates the sorting of tau proteins to lysosomes or late-endosomes (Wang et al., 2009). Cargo-loaded endosomes and lysosomes are transported to the cell periphery and fuse with the plasma membrane, releasing tau proteins into the extracellular space (Fontaine et al., 2016; Lee and Ye, 2018).

### Exosome/Ectosome-Based Secretion of Vesicular Tau

Intercellular communication can occur in multiple forms. With these diverse mechanisms, cells exchange their cellular information through the secretion and uptake of extracellular vesicles, which contain diverse cellular agents (proteins, lipids, reactive oxygen species, and genetic information) (Cocucci and Meldolesi, 2015). Mounting evidence has suggested that tau is one of the cellular components delivered by extracellular vesicles. The uptake of extracellular vesicles containing pathogenic tau aggregates can initiate tau pathology in recipient cells (Polanco et al., 2016; Wang et al., 2017). Extracellular vesicles are heterogeneous, with sizes ranging broadly from 50 to 1000 nm. Exosomes (30–150 nm) and ectosomes (100 and 1000 nm) are known to mediate tau transmission (DeLeo and Ikezu, 2018).

Exosomes are nano-sized vesicles derived from endosomal compartments called multivesicular bodies (MVBs) (Yáñez-Mó et al., 2015). Hsc70 delivers tau proteins into exosomes through interaction with the endosomal sorting complex required for transport (ESCRT) proteins (Wang et al., 2017). ESCRTs play a critical role in sorting exosomal cargos and secreting exosomes into the extracellular space. Once secreted into the extracellular space, exosomes are not only circulating in the brain-specific ISF, but also travel into the CSF and blood in humans (Caby et al., 2005; Fiandaca et al., 2015; Welton et al., 2017; Wu et al., 2017). Tau has been detected in the exosomes collected from the CSF and blood of AD patients (Saman et al., 2012; Jia et al., 2019). Particularly, in Jia et al. (2019) study, the level of exosomal tau was significantly higher in the CSF of AD patients than that of the healthy controls.

Ectosomes are microvesicles, released from the plasma membrane. Ectosome shedding is induced by intracellular calcium levels, inflammatory molecules, or oxidative stress (Piccin et al., 2007; Doeuvre et al., 2009). Ectosomal tau has also been found in neuroblastoma cell culture, the ISF of mice, and the CSF of AD patients and healthy controls (Dujardin et al., 2014; Spitzer et al., 2019). Recent evidence shows that neuron-derived exosomes or extracellular vesicles extracted from the AD patients had the potential to promote tau pathology when injected into the brains of wild-type mice (Winston et al., 2016; Ikezu et al., 2020).

## Molecular Mechanisms of Extracellular Tau Uptake

In the extracellular space, the level of tau is maintained by the balance between secretion and clearance. In peripheral organs, the lymphatic system mediates the clearance of cellular wastes in the ISF (Aspelund et al., 2015). However, in the brain, lymphatic vessels are found only in the brain meninges, not in the deep brain where tau aggregates are secreted. Instead, the glymphatic system has been highlighted due to its role in clearing extracellular wastes, including amyloid-β and tau, through paravascular ISF–CSF exchange (Iliff et al., 2012). Accordingly, the breakdown of the glymphatic system is associated with various neurodegenerative diseases, including AD and traumatic brain injury (Rasmussen et al., 2018). Microglia and astrocytes also play a role in eliminating extracellular tau (Bolós et al., 2016; Piacentini et al., 2017; Hopp et al., 2018). However, the prolonged activation of microglia and astrocytes could stimulate neuroinflammatory responses that facilitate neuronal degeneration. Therefore, microglia- and astrocyte-mediated clearance might be effective particularly at the early stage of AD (Kinney et al., 2018). In AD brains, extracellular tau interacts with amyloid precursor protein (APP) and β-amyloid. Takahashi et al. (2015) showed that the extracellular region of APP is involved in the uptake of tau fibrils into a cell.

Also, several studies have shown that the spreading of tau pathology was enhanced by the injection of β-amyloid into mouse brain. Actually, the binding affinity of tau for β-amyloid is almost 1000-fold higher than for tau (Guo et al., 2006). The mutual interaction between β-amyloid and tau exaggerates the cross-seeding effects of tau, spreading tau pathology in the brain (Vergara et al., 2019; Vogel et al., 2020). Extracellular tau proteins are also taken up by neurons *via* diverse cellular mechanisms. In recipient neurons, most of the internalized proteins are degraded through the endosome–lysosome route, but a portion of internalized tau could stimulate tau pathology, acting as a seed for native tau aggregation. In this section, we will review the diverse cellular mechanisms of tau uptake (Figures 1D,E).

### Heparan Sulfate Proteoglycan-Associated Macropinocytosis

Macropinocytosis is the bulk-endocytosis mediating the non-specific internalization of large amounts of extracellular fluid. Macropinocytosis plays a central role in tau pathology by mediating the internalization of large, insoluble tau aggregates (Wu et al., 2013; Evans et al., 2018). Tau aggregates bind to HSPGs on the neuronal surface, triggering macropinocytosis (Holmes et al., 2013; Rauch et al., 2018; Annadurai et al., 2021). Then, the extension of actin-dependent membrane ruffles engulfs tau aggregates, leading to the formation of macropinosomes. Macropinosomes are large vacuoles with an approximate diameter of up to 5 μm (Swanson, 2008). The maturation of macropinosomes involves membrane rearrangement for concentrating cargo proteins and then trafficking the cargos to the lysosome for degradation and recycling (Donaldson, 2019). The modification of HS chains on HSPGs is critical for tau binding and internalization. Rauch et al. (2018) reported that 6-O-sulfation of the HS sidechains is important for tau binding. Another study demonstrated that 3-O-sulfation enhances tau binding to the cell surface (Zhao et al., 2020).

### Receptor-Dependent Clathrin-Mediated Endocytosis

Clathrin-mediated endocytosis mediates the internalization of a wide range of cargo molecules from the cell surface to the interior. The process is characterized by the recruitment of cargo molecules by various transmembrane receptors and the formation of the clathrin-coated pit (CCP) for internalization (Doherty and McMahon, 2009). Recent studies have shown several endocytic receptors binding to extracellular tau. Lipoprotein receptor-related protein 1 (LRP1) (Lane-Donovan et al., 2014; Rauch et al., 2020), muscarinic acetylcholine receptors (mAChRs) (Morozova et al., 2019), and sorting protein-related receptor (SORLA) (Bok et al., 2021) are known to mediate tau internalization.

LRP1 is considered a major neuronal receptor that is involved in the internalization of extracellular tau. Rauch et al. (2020) reported that LRP1 is a master receptor mediating clathrin-mediated endocytosis of tau aggregates, playing a critical role in tau transmission. In their study, the lysine-rich microtubule binding domain of tau interacts with the cysteine-rich ligand binding domain of LRP1 (Rauch et al., 2020). The genetic silencing of LRP1 inhibited the uptake of various forms of tau, including monomers, oligomers, and fibrils, almost completely in H4 neuroglioma cells. The knockdown of LRP1 also suppressed the spreading of tau in the brain of mice expressing human tau P301L mutant. Cooper et al. (2021) also demonstrated LRP1-mediated internalization of tau aggregates extracted from AD patients. In their study, internalized tau monomers were degraded in lysosomes, but internalized tau aggregates induced tau seeding in the recipient cells. A member of the LDL-receptor superfamily, SORLA, was also identified as a receptor of extracellular tau (Cooper et al., 2021). mAChRs are also known to mediate clathrin-mediated internalization of tau. Gomez-Ramos et al. (2008)Gómez-Ramos et al. (2009) identified that the C-terminal domain of tau binds to M1/M3 subtypes of mAChR as a receptor agonist, increasing intracellular calcium concentration, and leading to neuronal cell toxicity. Morozova et al. (2019) demonstrated that mAChRs not only activate tau-induced calcium influx but also mediate the internalization of extracellular tau.

Recent genome-wide association studies (GWASs) have revealed genetic factors associated with sporadic AD. Specifically, APOE, bridging integrator 1 (BIN1), and phosphatidylinositol binding clathrin assembly protein (PICALM) showed a strong association with AD. PICALM binds to clathrin and its adaptor proteins, initiating CCP assembly (Meyerholz et al., 2005). Ando et al. (2016) reported the co-localization of phosphorylated tau with PICALM in the brains of AD patients. BIN1 is known to be involved in clathrin-mediated endocytosis through its interaction with clathrin and dynamin (Doherty and McMahon, 2009). However, the role of BIN1 in AD pathology remains largely unclear. Significantly decreased levels of BIN1 were observed in the brains of late-onset AD patients (Holler et al., 2014). The knock-down of BIN1 is known to promote endocytosis of extracellular tau aggregates (Calafate et al., 2016).

### Tau Clearance by Microglia and Astrocytes

Microglia, the professional phagocytes of the brain, mediates the clearance of extracellular tau aggregates, as well as dead or dying neurons bearing tau aggregates (Kofler and Wiley, 2011; Arcuri et al., 2017). In AD brains, extracellular tau proteins activate microglia, promoting phagocytosis of tau aggregates (Das et al., 2020). During this process, the extracellular tau binds CX3CR1, a chemokine receptor expressed on the surface of microglia (Bolós et al., 2016; Perea et al., 2020). Under physiological conditions, CX3CR1 interacts with fractalkines (CX3CL1), which are constitutively expressed by neuronal cells in either a membrane-bound or soluble form (Hatori et al., 2002). The CX3CR1/CX3CL1 axis plays a significant role in maintaining the homeostasis of the central nervous system. In the AD brain, the expression of fractalkines is reduced, disrupting the CX3CR1/CX3CL1 axis (Finneran and Nash, 2019). Additionally, tau aggregates compete to bind CX3CR1, mediating the phagocytosis of tau aggregates into microglia (Perea et al., 2020).

Astrocytes are specialized glial cells that are involved in the recycling and clearance of unnecessary synapses, synaptic debris, and extracellular protein aggregates in the brain (Sofroniew and Vinters, 2010; Jung and Chung, 2018). Astrocytes express a low amount of endogenous tau (Perea et al., 2019). However, elevated levels of tau have been observed in astrocytes of AD brains (Ferrer et al., 2018). Astrocytes are known to uptake extracellular tau proteins *via* distinct mechanisms depending on their aggregation states. Tau monomers are internalized by HSPGs-mediated translocation (Perea et al., 2019), and tau aggregates are internalized by endocytosis (de Calignon et al., 2012; Martini-Stoica et al., 2018). Tau aggregates bind to integrins on the surface of astrocytes, resulting in the activation of the integrin signaling pathway, which triggers tau endocytosis (Perea et al., 2020). In the early stage of tauopathies, the activation of astrocytes plays a critical role in neuroprotection, secreting pro-inflammatory cytokines and chemokines. However, prolonged activation of astrocytes eventually stimulates neuroinflammatory responses that facilitate neuronal degeneration (Zhang and Jiang, 2015; Ransohoff, 2016).

## Tunneling Nanotubes

Tunneling nanotubes are cell–cell communication bridges composed of filamentous-actin positive and tubulin negative membranous structures with a diameter of 50–800 nm (Davis and Sowinski, 2008). TNTs, discovered in 2004, indicate a novel mechanism for long-range intercellular communication in various cell types, such as immune cells and neuronal cells (Rustom et al., 2004). Recent *in vivo* imaging studies have shown the occurrence of TNTs in the mouse retina and cerebral cortex. Alarcon-Martinez et al. (2020) reported the occurrence of actin-positive TNTs between retinal pericytes using two-photon imaging. Chen and Cao (2021) reported actin-positive TNTs between astrocytes and neurons in the cerebral cortex of mice and showed EGFP transport from astrocytes to neurons in the cortex through TNTs. In addition, TNTs can be used as a pathway for transmitting various pathogens, including prions and prion-like proteins (Gousset et al., 2009). Among diverse prion-like proteins, tau is known as an extrinsic factor that increases TNT formation. Tardivel et al. (2016) showed that treatment of tau fibrils increased the TNT population in neuronal cells by almost three times. Additionally, Abounit et al. (2016) showed that tau fibril treatment increased TNT formation not only in neuronal cells but also in Hela cells. Based on these results, tau fibrils lead to an increase in TNT formation and, facilitate the intercellular spread of pathological tau.

## Conclusion

Tau pathology includes tau modification, aggregation, and transmission, which are associated with multiple cellular pathways. Accordingly, it is extremely challenging to identify therapeutic targets for tau-targeted drug discovery. During early trials, tau phosphorylation was considered as a key initiating event regulating tau pathology, and great effort was made for the development of kinase inhibitors. Unfortunately, all trials targeting kinases have failed to demonstrate clinical efficacy in patients with tauopathy, suggesting that tau phosphorylation is merely a part of tau pathology. There are also efforts to develop anti-tau agents inhibiting tau aggregation, or autophagy activators boosting the clearance of tau aggregates. More recently, extracellular tau is recognized as an important therapeutic target to prevent disease progression (Gomez-Ramos et al., 2006; Simon et al., 2012). Bepranemab, an anti-tau IgG4 antibody developed by Hoffmann-La Roche, is currently undergoing phase 2 clinical trials in AD patients (NCT04867616) (Albert et al., 2019). JNJ-63733657, an anti-tau IgG1 antibody developed by Janssen, is undergoing phase 2 trials in AD patients (NCT04619420) (Beshir et al., 2022). In the extracellular space, antibody-tau complexes are expected to be cleared as extracellular waste by microglia, astrocytes or the glymphatic system. However, the clearing efficiency of antibody-tau complexes has not been reported. So far, fragmented understandings of tau pathology cause repeated failures in tau-targeted drug discovery. We believe that comprehensive understanding of the fate of tau in physiological and pathological condition is necessary for the success of tau-targeted drug development.

## Author Contributions

SL, AS, KK, EF, and YK contributed to the search and assessment of the available literature. SL, AS, KK, and YK interpreted the results of previous studies and wrote the manuscript. SL, KK, YS, and YK edited and commented upon the final draft. All authors reviewed the manuscript.

## Conflict of Interest

The authors declare that the research was conducted in the absence of any commercial or financial relationships that could be construed as a potential conflict of interest.

## Publisher’s Note

All claims expressed in this article are solely those of the authors and do not necessarily represent those of their affiliated organizations, or those of the publisher, the editors and the reviewers. Any product that may be evaluated in this article, or claim that may be made by its manufacturer, is not guaranteed or endorsed by the publisher.
